# Hoarding Symptoms Are Not Exclusive to Hoarders

**DOI:** 10.3389/fpsyg.2016.01742

**Published:** 2016-11-10

**Authors:** Caterina Novara, Gioia Bottesi, Stella Dorz, Ezio Sanavio

**Affiliations:** ^1^Department of General Psychology, University of PadovaPadova, Italy; ^2^Casa di Cura Parco dei TigliPadova, Italy

**Keywords:** hoarding, clinical population, Bulimia, Binge eating, Major Depression, Anxiety Disorders, Psychosis

## Abstract

Hoarding disorder (HD) was originally conceptualized as a subcategory of obsessive compulsive disorder (OCD), and numerous studies have in fact focused exclusively on investigating the comorbidity between OCD and HD. Hoarding behavior can nevertheless also be found in other clinical populations and in particular in patients with eating disorders (ED), anxiety disorders (AD), major depression (MD), and psychotic disorders (PD). The current study was carried out with the aim of investigating, using a validated instrument such as the *Saving Inventory-Revised* (SI-R), the presence of HD symptoms in patients diagnosed with ED, AD, MD, and PD. Hoarding symptomatology was also assessed in groups of self-identified hoarders and healthy controls. The results revealed that 22.5% of the ED patients exceeded the cut-off for the diagnosis of HD, followed by 7.7% of the patients with MD, 7.4% of the patients with AD, and 5.9% of the patients with PD. The patients with ED had significantly higher SI-R scores than the other groups in the Acquisition and Difficulty Discarding scales while the AD, MD, and PD patients were characterized exclusively by Difficulty Discarding. These data suggest to clinicians that hoarding symptoms should be assessed in other types of patients and especially in those affected by Bulimia and Binge eating.

## Introduction

Hoarding disorder (HD) has recently been included in Obsessive-compulsive spectrum disorders of DSM-5 as a distinct condition (Mataix-Cols et al., 2010; Pertusa et al., 2010). From the time studies were first carried out on the disorder (Frost and Gross, 1993; Frost and Hartl, 1996) HD has been defined and characterized by: (a) excessive acquisition of large quantities of useless objects; (b) difficulty in discarding possessions; (c) cluttering of living spaces so as to preclude the activities for which they were intended. It has been reported that after onset, HD generally follows a chronic course with prevalence ranging from 2.3% in younger age groups to 6.2% in older ones (Samuels et al., 2008) and spontaneous remissions are rare (Gilliam and Tolin, 2011). According to a study by Italian investigators, its prevalence reaches as high as 6% in the Italian population (Bulli et al., 2014). The prevalence of hoarding in the female gender is controversial: in a sample of self-identified hoarders (SIH), Frost et al. (2011) reported that 78% was made up of women while the percent of women and men in the general population was, respectively, 5.6 and 2.6%. No gender differences were found in the non-clinical Italian population (Bottesi and Novara, 2012; Bulli et al., 2014).

The problem of comorbidity has always characterized hoarding because of a poor diagnostic definition (until DSM-5) and the symptoms overlap with those observed in mood disorders, anxiety disorders (AD), eating disorders (ED), or Schizophrenia Spectrum. The perspective has generally been unidirectional when other psychopathological characteristics have been evaluated in HD patients and in some cases bidirectional when HD characteristics have been investigated in patients diagnosed with other disorders. Absence of relevant comorbidity has been reported in 25 to 42% of hoarders (London Field Trial for HD; Grisham et al., 2005; Frost et al., 2011; Mataix-Cols et al., 2012; Hall et al., 2013).

### Comorbidity with Depression and Anxiety

Since HD has been considered a subcategory of obsessive compulsive disorder (OCD), several studies have considered samples of hoarders exclusively with OCD comorbidity. Although according to some studies approximately 18–33% (Rasmussen and Eisen, 1989; Frost and Hartl, 1996) of OCD patients have hoarding comorbidity, 83% percent of hoarders do not meet the criteria of OCD (Frost et al., 2010). A stronger comorbidity with Mood Disorders [Major Depressive Disorder (MDD) 50–75%] and AD [Generalized Anxiety Disorder (GAD) 23–39%, Panic 15%, Agoraphobia 14%, Specific phobia 26–29%, and Social phobia 14–71%; Samuels et al., 2002; Lochner et al., 2005] has, moreover, been found in OCD patients with HD. The fact that depression is the most commonly co-occurring symptom in HD has also been confirmed by a recent study designed by Hall et al. (2013) who reported that 42% of the self-identified HD population had depression as measured by the Depression and Anxiety Stress Scale (DASS; Lovibond and Lovibond, 1995).

One of the few studies that considered diagnoses other than OCD is one by Tolin et al. (2011) who examined hospitalized patients presenting for treatment of anxiety problems. That study uncovered that 28% of the GAD patients had clinically significant hoarding symptoms followed by the patients diagnosed with OCD (16.6%) and those with Social Phobia (14.8%). These data have also been confirmed in a population diagnosed as hoarders in whom the prevalence of MD reached 50.7%, followed by GAD (24.4%), Social Phobia (23.5), and OCD (18%) (Frost et al., 2011).

While comorbid hoarding symptomatology in OCD populations is well established and has been amply demonstrated, it is not clear if it is linked to anxiety or depression given the scarcity of studies carried out until now. The current study, in fact, aimed to investigate hoarding symptomatology in groups of those patients.

### Comorbidity with Eating Disorders

The association between HD and ED is controversial and understudied. When OCD hoarders were compared with OCD non-hoarders, the severity of symptoms seemed to depend on the female component of the sample which also included ED patients (Wheaton et al., 2008). Some of the first studies (Halmi et al., 2003) that examined hoarding in ED patients found that those subjects did not differ from OCD controls as far as presence of hoarding symptoms was concerned (16.5% vs. 13.8%, respectively). This finding was recently confirmed by an Italian study which reported that 15.5% of the ED patients studied exceeded the clinical cut-off level (Novara and Bottesi, 2013). Fontenelle et al. (2004) reported, moreover, that 26.7% of the hoarders also had an ED (Bulimia in 13.3% of the cases and Binge eating in the rest), while Frost et al. (2011) found that only 1.4% of a population of hoarders had ED.

Until now a single item on the Yale-Brown Obsessive Compulsive Scale (Y-BOCS; Goodman et al., 1989) specifically concerned with hoarding has been used to investigate hoarding symptomatology in ED patients. The current study, which assessed these patients using a more exhaustive, specific measure such as the Saving Inventory-Revised (SI-R), will make it possible to compare its findings with those that will be registered by future investigations based on standardized assessment instruments.

### Comorbidity in Psychosis

The relationship between HD and psychosis has been studied less assiduously although it is considered one of the pathologies associated to *Hoarding* (Pertusa et al., 2010). Repetitive acquisition behaviors and excessive care of objects of little value have often been found in patients with schizophrenia and have been positively correlated especially to the male gender and the Caucasian race (Luchins et al., 1992). In a study carried out to quantify the presence and the variety of repetitive behaviors (including hoarding) in a sample of 400 chronic patients with schizophrenia, Tracy et al. (1996) reported that the percent of patients manifesting abnormal acquisition behavior was below 20%. No other study, to our knowledge, has specifically investigated HD characteristics in patients with schizophrenia.

The principal aim of the current study was to investigate the prevalence of HD symptoms in patients diagnosed with ED, Major Depression (MD), AD, and Psychosis (PD) as well as in groups of SIH and healthy controls using a validated, standardized assessment instrument such as the SI-R. Given literature findings, we expected to identify a greater frequency of depression symptoms in a group of SIH (SIH group) and the highest percentage of hoarding symptoms in the AD patients. The study also intended to examine the relationship between HD symptoms severity and disease duration.

## Materials and Methods

### Participants

One hundred twenty-four patients with an established diagnosis were enrolled in the study. Forty-one (100% female) of these were consecutively hospitalized patients suffering from Bulimia or Binge eating (ED group = 10 had the former and 31 the latter); the others were consecutively presenting outpatients who were grouped as follows: 39 (56.4% female)had MD (MD group), 27(63% female) had AD (AD group = 17 with general anxiety disorder, 6 with panic disorder, 2 with adjustment disorders with anxiety, 1 with acute stress disorder, 1 with social AD), 17 (52.9% female) had psychotic disorders (PD group in the recovery phase = 12 were diagnosed with schizophrenia and 5 with schizoaffective disorder). All of the patients were diagnosed by expert clinicians following the Diagnostic and Statistical Manual of Mental Disorders - Fourth Edition - Text Revision (DSM-IV-TR; American Psychiatric Association [APA]). Pharmacotherapy regarded 36,58% of ED group (SSRI or phenothiazine), 46,15% of MD group (SSRI), 35,29% of AD group (SSRI), and the 52,94% of PD group (clozapine or valproate sodium).

Exclusion criteria to the study were: mental retardation, head injury/neurologic diseases, and symptoms that could be signs of serious health conditions. Patients with comorbid personality disorders were not excluded (ED group: *N* = 6; MD group: *N* = 3; AD group: *N* = 3; PD group: *N* = 1). This study was approved by the Ethical Committees of the Department of General Psychology and by the clinical structure in which the participants were hospitalized and/or interviewed. The 49 (83.7% female) SIH who were enrolled were considered the SIH group; 48 (66.7% female) healthy individuals from the community at large were also enrolled (and considered the healthy control – HC-group).

### Instruments

The Italian version of the SI-R (Frost et al., 2004; Novara et al., 2013), a 23-item self-report questionnaire which quantifies compulsive hoarding, was used to assess hoarding in all of the groups studied. The respondents were asked to rate the items on the inventory’s three sub-scales – Clutter, Difficulty Discarding/Saving, and Acquisition – with reference to situations taking place over the previous week’s time using a 5-point Likert scale (0 = never, 1 = rarely, 2 = sometimes/occasionally, 3 = Frequently/Often, 4 = Very often). In addition to a total score, it is also possible (for both the original and the Italian versions) to compute sub-scores for each of the sub-scales; these were found to show good internal consistency and test-retest reliability. Scores exceeding 40 indicate the presence of clinically significant HD. The following cut-offs scores were identified for the original English version: Clutter: raw scores ≥ 15; Difficulty discarding: raw scores ≥ 13; Acquisition: raw scores ≥ 13 (Frost et al., 2004).

The *Beck Anxiety Inventory* (BAI; Beck et al., 1988; Italian version Sica et al., 2006), which is a 21-item multiple-choice self-report inventory, was used to measure the severity of anxiety in the participants. The items inquire about common symptoms recently experienced by the respondent; the inventory has been found to have a good internal consistency and test–retest reliability. Good psychometric properties have also been confirmed for the Italian version.

The *Beck Depression Inventory – II* (BDI-II; Beck et al., 1996; Italian version Ghisi et al., 2006), which is a 21-item multiple-choice self-report inventory and one of the most widely used psychometric tests, was utilized to evaluate depression in the participants. The instrument identifies the presence and severity of affective, cognitive, motivational, psychomotor, and vegetative symptoms of depression in accordance with the Diagnostic and Statistical Manual of Mental Disorders (DSM-IV) criteria. The original version of the BDI-II was found to have good internal consistency (both in students and in patients), good test-retest reliability at 1 week, and good convergent and discriminant validity. The Italian version of the BDI-II was also found to have good psychometric properties.

### Procedure

The overall recruitment was carried out between 2013 and 2015 by experienced operators; no incentive was offered for participation. The individuals with ED were consecutively hospitalized patients; the individuals with MD, AD, and PD were outpatients recruited at the mental health units specialized in the treatment of those psychopathologies. The SIH were respondents to advertisements on university bulletin boards and Internet sites seeking volunteers for a research study examining HD features in the Italian population. The large pool of individuals (*N* = 515) who voluntarily agreed to complete a battery of questionnaires were informed about the aims of the research and were asked to sign consent statements. Those volunteers who got a score higher than the 90° percentile on the total SI-R score (raw score ≥ 36) were enrolled in the SIH group. The healthy subjects included in the HC group were likewise individuals who responded to advertisements seeking volunteers for psychological studies.

All the patients in the ED, AD, MD, and PD groups and the individuals in the SIH and HC groups received a complete explanation of the study’s aim and what their participation entailed. All signed informed consent statements to participate in the study. The participants were asked to complete a form providing their socio-demographic details. They were then asked to fill out the three inventories which were administered in a counterbalanced order to avoid any order effects.

### Statistical Analyses

Pearson’s chi square and univariate analysis of variance (ANOVA) were used to compare groups with regard to socio-demographic variables and the BAI, BDI-II, and SI-R scores; the Bonferroni *post hoc* procedure was used to further explore mean differences between groups.

In order to further assess group and gender differences on the SI-R scores, a series of 2 (group) × 2 (gender) ANOVAs was conducted.

Cronbach’s alphas were computed for each group’s total and subscale SI-R scores in order to assess the internal consistency of the questionnaire. The internal consistency values were thus rated: α ≥ 0.90 = “optimal”; 0.90 > α ≥ 0.80 = “excellent”; 0.80 > α ≥ 0.70 = “good”; 0.70 > α ≥ 0.60 = “sufficient”; α < 0.60 = “insufficient.”

Pearson’s correlations were conducted separately for the ED, MD, AD, and PD groups in order to explore the association between disease duration, age, and total SI-R scores.

## Results

Means, standard deviations (SD) ranges and Bonferroni *post hoc* comparisons with reference to age, education, disorder duration, and BAI and BDI-II scores are outlined in **Table 1**.

**Table 1:** Means standard deviations (*SD*) ranges and *post hoc* comparisons of age, education, duration of the disorder, BAI and BDI-II scores across the groups studied.

	ED	MD	AD	PD	SIH	HC	Bonferroni
	(*N* = 41)	(*N* = 39)	(*N* = 27)	(*N* = 17)	(*N* = 49)	(*N* = 48)	*post hoc*
**Age (yr)**	20.59	49.95	45.19	52.24	25.02	36.27	MD=AD=PD=HC>ED
	(4.34)	(15.25)	(15.85)	(11.51)	(9.26)	(15.23)	MD = AD = PD = HC>SIH
	16–35	18–78	19–74	34–70	20–50	23–60	
**Education (yr)**	11.46	11.28	12.89	10.29	11.94	14.13	ED=MD=PD<HC
	(3.52)	(3.22)	(3.52)	(3.18)	(3.07)	(3.49)	
	8–25	5–18	7–20	5–14	8–18	8–21	
**Duration of thedisorder (yr)**	4.00	10.11	10.67	17.65	–	–	ED<PD MD=AD=PD
	(2.83)	(10.43)	(10.13)	(11.39)			
	1–10	1–40	0–40	1–40			
**BAI**	8.30	7.19	9.92	3.87 (4.62)	10.74	8.87	–
	(9.29)	(7.63)	(11.90)		(7.55)	(6.75)	
	0–42	0–33	0–53	0–16	0–29	0–29	
**BDI-II**	20.77	23.77	16.63	22.35	29.61	9.18	ED=AD<SIH
	(12.77)	(9.97)	(10.55)	(11.28)	(6.45)	(7.67)	MD=PD=SIH>HC
	0–43	6–45	0–38	8–48	16–45	0–31	

*ED, eating disorders; MD, major depression; AD, anxiety disorders; PD, psychotic disorders; SIH, self-identified hoarders; HC, healthy controls; BAI, Beck Anxiety Inventory; BDI-II, Beck Depression Inventory -II.*

The BAI (Beck et al., 1988; Italian version Sica et al., 2006) uncovered no differences between groups as far as physiological anxiety symptoms were concerned (*F*_5,215_ = 2.05, *p* = 0.07). There were instead significant differences between groups in depression symptom scores on the BDI-II (Beck et al., 1996; Italian version Ghisi et al., 2006) (*F*_5,215_ = 24.01, *p* = < 0.001). Bonferroni *post hoc* comparisons revealed that the healthy controls had significantly lower scores with respect to the other groups (all *p*s < 0.05). While the SIH group had higher scores than the ED (*p* > 0.001) and AD (*p* > 0.001) groups, no other differences between groups were found. Mean group SI-R scores are outlined in **Table 2**.

**Table 2:** Means, standard deviations (SD), ranges and Bonferroni *post hoc* comparisons for the Saving Inventory-Revised (SI-R) Total and subscales scores obtained by the 6 groups.

	ED group	MD group	AD group	PD group	SIH group	HC group	*F*_5,215_	*p*	Bonferroni
	(*N* = 41)	(*N* = 39)	(*N* = 27)	(*N* = 17)	(*N* = 49)	(*N* = 48)			*post hoc*
**Clutter**	5.85	5.67	5.81	7.18	15.61	6.40	23.92	<0.001	ED=MD=AD=PD=HC<SIH
	(6.04)	(5.76)	(5.59)	(5.56)	(5.02)	(4.72)			
	(0–22)	(0–22)	(0–22)	(0–17)	(8–33)	(0–20)			
**Difficulty discarding**	9.55	7.61	9.33	9.82	17.45	8.65	23.20	<0.001	ED=MD=AD=PD=HC<SIH
	(6.26)	(5.10)	(4.75)	(6.34)	(4.01)	(4.30)			
	(0–21)	(0–25)	(3–18)	(1–26)	(9–28)	(2–17)			
**Acquisition**	10.76	6.95	7.37	7.71	15.06	6.98	20.76	<0.001	ED,MD,AD,PD,HC<SIH
	(6.50)	(4.61)	(4.43)	(4.22)	(3.99)	(3.94)			MD=HC<ED
	(0–26)	(1–20)	(3–22)	(2–17)	(7–26)	(0–19)			ED=AD=PD
**Total**	25.87	20.23	22.52	24.71	48.12	22.02	33.17	<0.001	ED=MD=AD=PD=HC<SIH
	(15.87	(13.22)	(13.05)	(12.85)	(8.52)	(10.61)			
	) (3–63)	(2–62)	(6–60)	(9–44)	(38–67)	(4–53)			

*ED, eating disorders; MD, major depression; AD, anxiety disorders; PD, psychotic disorders; SIH, self-identified hoarders; HC, healthy controls.*

Bonferroni *post hoc* comparisons revealed that the SIH group had significantly higher Clutter scale scores with respect to the other groups (all *p*s < 0.001); the scores of the other groups were comparable (all *p*s > 0.05). A similar pattern was likewise found in the Difficulty Discarding scale as the SIH group had significantly higher scores than all the other groups (all *p*s < 0.001), the scores of the other groups were comparable (all *p*s > 0.05). Finally, with regard to the Acquisition scale, the SIH group had higher scores with respect to all the other groups (all *p*s < 0.001). The ED group had scores that were higher than the MD and HC groups (*p* = 0.006 and *p* = 0.003, respectively); there were no differences between the ED, AD, and PD groups (all *p*s > 0.05). Finally, the SIH group had significantly higher total SI-R scores (than all the other groups all *p*s < 0.001); the scores of all the other groups were comparable (all *p*s > 0.05).

Results emerged from the 2 (group) × 2 (gender) ANOVAs revealed a significant main effect of gender in regard to the Difficulty Discarding scale (*F*_1,219_ = 6.33; *p* = 0.01): in particular, males (*M* = 11.57; *SD* = 6.35) scored higher than females (*M* = 10.41; *SD* = 6.06). Furthermore, a significant group × gender interaction (*F*_4,219_ = 2.70; *p* = 0.03) emerged: specifically, in the PD group males obtained significantly higher scores than females (*p* = 0.02), whether no gender differences emerged within the other groups (all ps > 0.05) A significant main effect of gender emerged also in regard to the total SI-R score (*F*_1,219_ = 5.03; *p* = 0.03); also in this case, males (*M* = 30.26; *SD* = 17.41) scored higher than females (*M* = 27.71; *SD* = 15.61).

Internal consistency values for the SI-R ran from sufficient (α = 0.67). To optimal (α = 0.95) for all the groups assessed.

On the basis of the wide range of score obtained by the different groups, it was decided to analyze the questionnaires from a categorical point of view.

### Symptom Severity in the Study Groups

The percentages of participants with scores on the SI-R (total and subscales) above the cut-off levels are shown in **Table 3**.

**Table 3:** Number and (percentages) of individuals in the six groups assessed scoring above the cut-off levels.

	ED group	MD group	AD group	PD group	SIH group	HC group
	(*N* = 41)	(*N* = 39)	(*N* = 27)	(*N* = 17)	(*N* = 49)	(*N* = 48)
**Total SI-R**	9 (22.5)	3 (7.7)	2 (7.4)	1 (5.9)	39(79.6)	2 (4.2)
**Clutter**	5 (12.2)	5 (12.8)	2 (7.4)	2 (11.8)	24 (49)	2 (4.2)
**Difficulty discarding**	12 (30)	7 (17.9)	7 (25.9)	6 (35.3)	45 (91.8)	9 (18.8)
**Acquisition**	13 (31.7)	5 (12.8)	2 (7.4)	3 (17.6)	36 (73.5)	5 (10.4)
**BAI**	4 (9.8)	4 (10.8)	5 (18.5)	1 (6.3)	11 (23.4)	8 (17)
**BDI II**	20 (51.3)	21 (53.8)	10 (37)	8 (47.1)	45 (91.8)	4 (8.3)

*ED, eating disorders; MD, major depression; AD, anxiety disorders; PD, psychotic disorders; HD, hoarding disorder; HC, healthy controls.*

Study findings highlighted significant differences between groups with regard to the percentage of individuals scoring above the cut-off level on the SI-R total score (χ_5_^2^ = 101.88, *p* < 0.001) as well as on the Clutter (χ_5_^2^ = 42.05 *p* < 0.001), Difficulty Discarding (χ_5_^2^ = 76.38, *p* < 0.001), and Acquisition (χ_5_^2^ = 67.45, *p* < 0.001) scale scores. Just as in other studies examining groups of patients diagnosed with hoarding, those individuals included in the SIH group studied here had higher percentages of scores above the cut-off levels on all the scales with respect to the other groups. The percentages of individuals in the ED and MD groups with scores above the cut-off levels on the Clutter scale were quite similar (12.2 and 12.8%, respectively); those percentages were higher than those in the other groups (ranging from 4.2 and 11.8%). The percentages of individuals in the ED and PD groups with scores above the cut-off levels on the Difficulty Discarding scale were quite similar (30 and 35.3%, respectively) and were higher than those observed in the other groups (range: 17.9–25.9%). Finally, the percentage of ED patients who had scores above the cut-off levels on the Acquisition scale was higher (31.7%) than that in the other groups (percentages ranging from 7.4 to 17.6%), but lower than that in the HD group (73.5%).

There were no differences between groups with regard to the percentages of individuals who had scores above the BAI cut-off levels (χ_5_^2^ = 5.25, *p* = 0.39); only 15.3% of the entire sample had clinically significant scores on this measure. There were instead significant differences with regard to the BDI-II (χ_5_^2^ = 69.74, *p* < 0.001). A higher percentage (91.8%) of the individuals included in the SIH group had scores above the cut-off level with respect to the other groups (percentages ranging from 8.3 to 53.8%).

### Associations between Age, Duration of the Disorder, and Hoarding Symptoms

Correlational analyses did not uncover any associations between age or disease duration and the total SI-R score in any of the groups (ED: *r* = 0.08/12; MD: *r* = 0.20/-0.16; AD: *r* = -0.13/0.34; PD: *r* = 0.09/.19; SIH: *r* = 0.04).

## Discussion

None of the MD, AD, PD, or ED patients had sought assistance for problems linked to hoarding. Contrary to our hypothesis, 22.5% of the patients diagnosed with Bulimia and Binge eating exceeded the clinical cut-off levels for hoarding symptomatology; 7.7% of the MD, 7.4% of the AD, and 5.9% of the PD also did so. The fact that the study only considered patients with Bulimia and Binge eating and that a specific assessment measure was used to evaluate hoarding may explain the high percentage of hoarding symptoms found with respect to other studies whose data (Halmi et al., 2003) were based on a single item on the Y-BOCS (Goodman et al., 1989) specifically concerned with hoarding. Our ED group was moreover composed entirely of hospitalized patients and this could further explain the high percentage found with respect to the other groups. In light of these results, the higher prevalence reported for HD symptoms in ED might seems not to depend on a higher prevalence of females with a generally severe clinical condition requiring inpatient treatment.

As far as the patients with MD (7.7%) and PD (5.9%) were concerned, no comparison can be made with data in the literature as no studies have been carried out on these populations, and assessment of hoarding, in any case and as has already been mentioned, has always been evaluated using a non-specific instrument (Tracy et al., 1996). The total percent of hoarders in our AD sample was inferior to that reported in the literature (Tolin et al., 2011), and this might be explained by differences in disease severity at the time of the initial diagnosis. Our sample was, for example, entirely made up of non-hospitalized patients while Tolin et al. (2011) study included only hospitalized ones.

When single subscales were considered, approximately 30% of the ED patients exceeded the clinical cut-off level with regard to both the Difficulty Discarding and Acquisition scales while Difficulty Discarding was the most representative symptomatology in the other groups of patients (35.3% for PD; 25.9% for AD; 17.9% for MD). The different clinical diagnosis considered (ED, PD, AD, MD) were thus characterized by single elements of the HD symptomatology: patients with Bulimia and Binge Eating had more Acquisition and Difficulty Discarding problems, while the PD, AD, and MD had only more Difficulty Discarding problems.

When the groups were compared utilizing the SI-R as a continuous variable, only the ED patients had significantly higher scores on the Acquisition scale with respect to the depression group; the findings for the rest of the clinical groups and the control patients, with the exception of the SIH, were comparable. These data confirm precedent studies with regard to construct independence and the diagnosis of HD defined by its characteristics of Clutter, Acquisition and Difficulty Discarding, (Frost and Hartl, 1996) but they also underline the importance of investigating specific features of hoarding in specific groups: Acquisition and Difficulty Discarding should be assessed, in particular, patients with Bulimia and Binge Eating, while Difficulty Discarding should be evaluated in patients with AD, MD, and PD.

As expected, depression was the most common comorbid condition in the SIH group; 91.8% of our sample exceeded the clinical cut-off level for depression and the BDI score registered by that group was similar to that in the depressed group. That percentage was found to be higher than ones registered by precedent studies examining individuals with HD selected from the general population (Frost et al., 2011) even if the individuals with SIH evaluated by our study had in fact SI-R scores that were overlapping with those of other HD (Frost et al., 2004) subjects. This may have been due to a characteristic of the Italian population that has not yet been extensively studied or to a self-selection bias on the part of the SIH who were enrolled in the study; we cannot, in fact, exclude the presence of other clinical symptoms in that group of participants.

No association was found between hoarding severity, disease duration and age in the clinical groups. According to our findings, when hoarding is a comorbid condition with other pathologies, it does not seem to worsen when age increases. It is possible that the symptoms are stable and ego-syntonic and need to be investigated more extensively during the initial assessment phase.

One of the study’s principal limitations concerns the persons in the SIH group. As they did not complete other questionnaires investigating general or specific psychopathologies such as OCD, it is impossible to exclude other concomitant pathologies. The prevalence of hoarding found in our group, which was equal to 7.57% (39/515), was nevertheless consistent with percentages in the general population registered by other studies (2–6 %; Samuels et al., 2008; Bulli et al., 2014). Given the limitations of self-report inventories, we are convinced that only an appraisal of a patient’s habitation or a personal interview with the patient him/herself or a relative can confirm a diagnosis of hoarding, a disorder which is frequently underestimated by clinicians. Furthermore, the higher prevalence of females than males in all the groups, despite apparently not affecting our results, might limit the generalizability of present findings. Another obstacle to generalizability might regard the medication regimen of participants; for example, some evidence suggests that hoarding behavior in Schizophrenia might depend on antipsychotic treatment (Pertusa and Fonseca, 2014).

These data suggest that hoarding symptoms should also be investigated in other types of patients and especially in those affected with Bulimia, Binge eating, AD, MD as well as in subjects with psychosis in remission. Future studies should consider also the role of emotional regulation in the development and maintenance of hoarding symptoms in clinical populations, since it has resulted as a mediating variable in ED (Raines et al., 2015) as well as in HD (Shaw et al., 2015).

## Author Contributions

CN: Conducted experiments and wrote the manuscript. GB: Contributed to the statistical analysis. SD: Contributed to the recruitment of patients. ES had an organizational role.

## Conflict of Interest Statement

The authors declare that the research was conducted in the absence of any commercial or financial relationships that could be construed as a potential conflict of interest.
